# The Role of Dupilumab in Atopic Dermatitis-Like Graft-Versus-Host Disease

**DOI:** 10.7759/cureus.88293

**Published:** 2025-07-19

**Authors:** Inês Ferreira Costa, Diana Pinto, Fernanda Teixeira, Iris Maia, Rosa Branca Ferreira, Isabel Inácio, Ana Rita Araújo

**Affiliations:** 1 Pediatric Allergology Unit, Centro Materno Infantil do Norte, Unidade Local de Saúde de Santo António, Porto, PRT; 2 Pediatrics, Unidade Local de Saúde de Santo António, Porto, PRT; 3 Pediatric Oncology, Instituto Português de Oncologia do Porto Francisco Gentil, Porto, PRT

**Keywords:** atopic dermatitis, children, dupilumab, graft vs host disease, quality of life (qol)

## Abstract

Atopic dermatitis-like graft-versus-host disease (AD-like GVHD) is a rare but challenging complication following hematopoietic stem cell transplantation (HSCT), mimicking features of atopic dermatitis (AD) and often requiring prolonged immunosuppression. We report the case of a 14-year-old girl with a history of mild AD and post-HSCT GVHD involving skin and the gastrointestinal tract, presenting with severe pruritus, extensive eczema, and significant impact on quality of life. Standard treatment with corticosteroids and immunosuppressants yielded suboptimal results and notable side effects, including growth retardation. After multidisciplinary evaluation, off-label treatment with dupilumab was initiated, resulting in significant clinical improvement, corticosteroid tapering, and enhanced quality of life. This case highlights the potential role of dupilumab as a safe and effective corticosteroid-sparing therapy for AD-like GVHD in pediatric patients, although further studies are needed to confirm its long-term safety and efficacy in transplant settings.

## Introduction

Atopic dermatitis-like graft-versus-host disease is a skin condition that occurs after hematopoietic stem cell transplantation, first reported by Tanasescu in 1999 [1]. Clinical characteristics of AD-like GVHD include generalized erythema, scaling, xerosis, and significant pruritus. Distinguishing this entity from classic AD can be challenging, as they share similar clinical and histologic features. In both conditions, T-helper (Th) cell-driven immune responses play a central role. Specifically, Th2-mediated inflammation is predominant, characterized by elevated interleukin (IL)-4, IL-5, and IL-13, leading to eosinophilia, IgE overproduction, and skin barrier dysfunction. This immunological overlap may contribute to the AD-like phenotype seen in certain GVHD presentations. In severe cases, both AD and AD-like GVHD may require long-term immunosuppression. However, chronic corticosteroid use is associated with significant adverse effects and a reduced quality of life, highlighting the need for new, targeted therapies that are both safe and effective [2,3].

This clinical case was previously presented as an oral presentation at the EIP 2023 - 15th Excellence in Pediatrics Conference, on December 1, 2023.

## Case presentation

**Figure 1:**
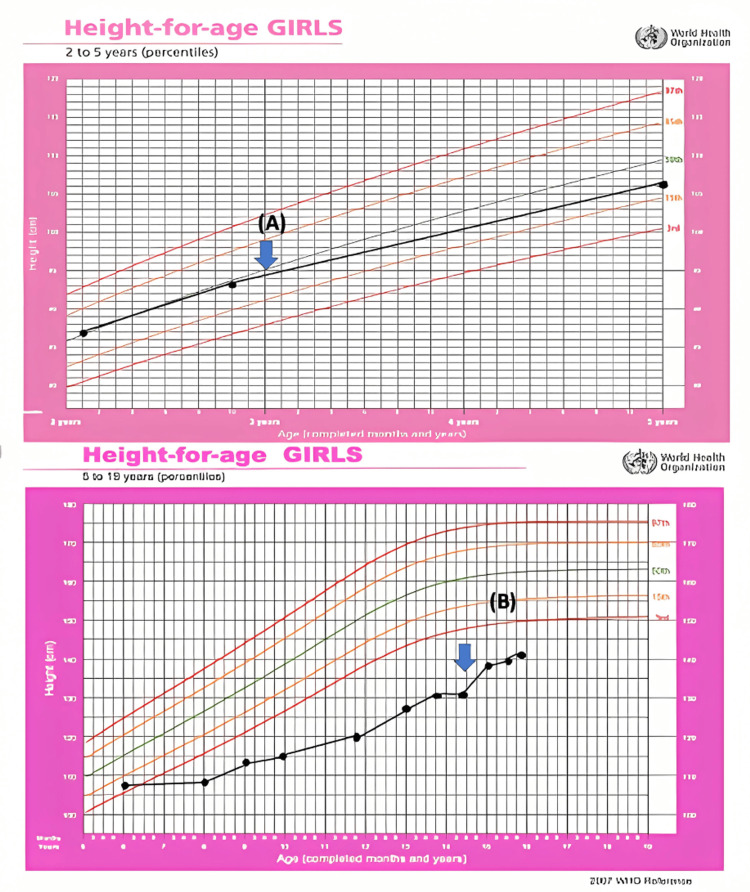
WHO height chart for age, with height marked with black dots, beginning of the disease marked with arrow (A), and beginning of treatment with dupilumab marked with arrow (B). Adapted from WHO’s Growth Standards Chart.

We report a case of a 14-year-old girl with mild AD since the first six months of life, optimized with topical treatment. She was diagnosed with bone marrow aplasia at the age of three years old and received an allogeneic HSCT. Six months after that, she developed cutaneous followed by intestinal GVHD. She presented with debilitating generalized pruritus, highly limiting her ability to sleep, and skin lesions involving approximately 45% of the total body surface area, particularly affecting the face, back, and extremities. The lesions were characterized by erythema, dry skin, and intense itching. Additionally, she had abdominal pain, diarrhea, and incoercible vomiting, which led to hospitalizations due to oral intolerance and dehydration. Laboratory tests revealed eosinophilia (640/mL), increased total IgE (3040 kU/L), and sensitization to multiple food and aeroallergens. She was advised to follow a restrictive diet, avoiding milk, eggs, some fruits, and shellfish, with no improvement. Given the severity of the presentation, she started treatment with topical and systemic corticosteroids (prednisolone 1 mg/kg/day), calcineurin inhibitors, and oral antihistamines, without achieving full resolution. Despite the slight cutaneous improvement, she often had flare-ups, and side effects of long-term corticosteroid therapy became apparent (cushingoid appearance, weight gain, and short stature). The severity of clinical features, as measured by a disease activity score, indicated moderate to severe AD (Scoring Atopic Dermatitis [SCORAD] = 59.4) [4,5], with a negative impact on quality of life. Because of the severe itching, the patient and her parents reported social isolation, dysthymic humor, and only a few hours of sleep each day. This sleep deprivation, along with the use of systemic corticosteroids, compromised her growth, causing a drop across three percentile curves (from the 50th-85th percentile to below the third percentile for girl height) (Figure 1).

**Figure 2:**
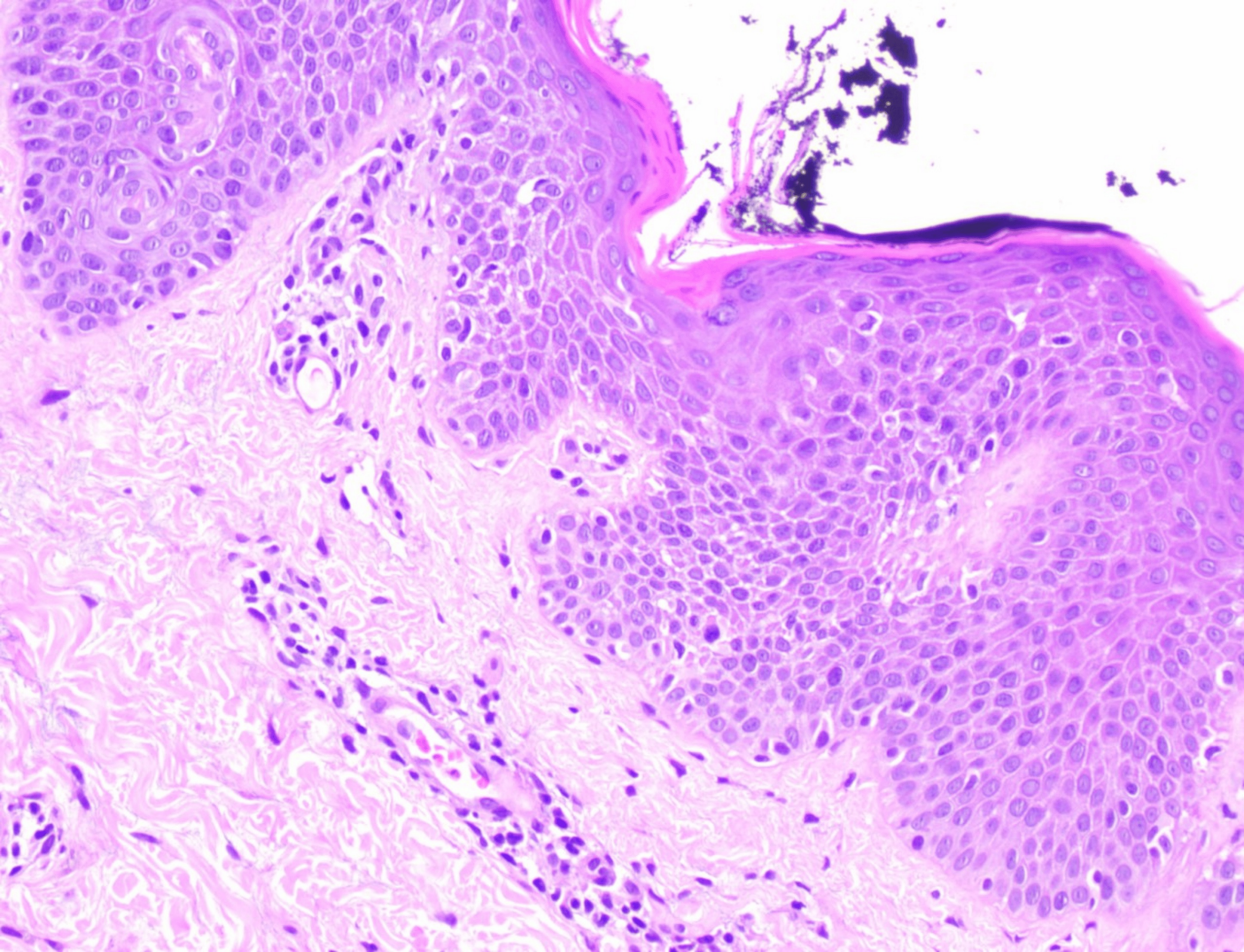
A skin biopsy revealed subacute spongiotic dermatitis, hyperkeratosis, acanthosis, and a perivascular mild lymphohistiocytic infiltrate with an absence of eosinophils, confirming the diagnosis. (Hematoxylin-eosin stain; original magnification: ×200).

After a multidisciplinary discussion (dermatology, pediatric allergy, oncology, and bone marrow transplantation physician), it was decided to reduce corticosteroid therapy, and at 11 years old, a skin biopsy revealed subacute spongiotic dermatitis with a perivascular mild lymphohistiocytic infiltrate (Figure 2).

At the age of 12, she began treatment with a luteinizing hormone-releasing hormone (LHRH) analogue, which she continued for a year, in order to delay the closure of the bone epiphyses and maximize her final height. 

Given the lack of a satisfactory response to standard therapy and the risk of significant side effects with long-term systemic corticosteroids, the risks vs. benefits of dupilumab were considered, and the medical team proposed to use dupilumab off-label.

Following approval by the institutional ethics and pharmaceutical commissions, informed consent was obtained from the patient’s parents, and assent was provided by the adolescent. The patient initiated treatment with a loading dose of 400 mg of subcutaneous dupilumab, followed by 200 mg every two weeks. After four months of therapy (total cumulative dose: 2,000 mg), she showed a marked clinical improvement, with a significant reduction in pruritus and inflammation. This allowed for a gradual tapering of systemic corticosteroids over the subsequent seven months, ultimately leading to their complete discontinuation. Clinically, the number and extent of active skin lesions decreased substantially, with residual eczema limited to the popliteal region and minimal excoriations. Although complete remission was not achieved, near-complete resolution was observed by month seven. In addition to these objective improvements, the patient and her caregivers reported a significant enhancement in quality of life, including better sleep, improved mood, and re-establishment of social engagement.

**Figure 3:**
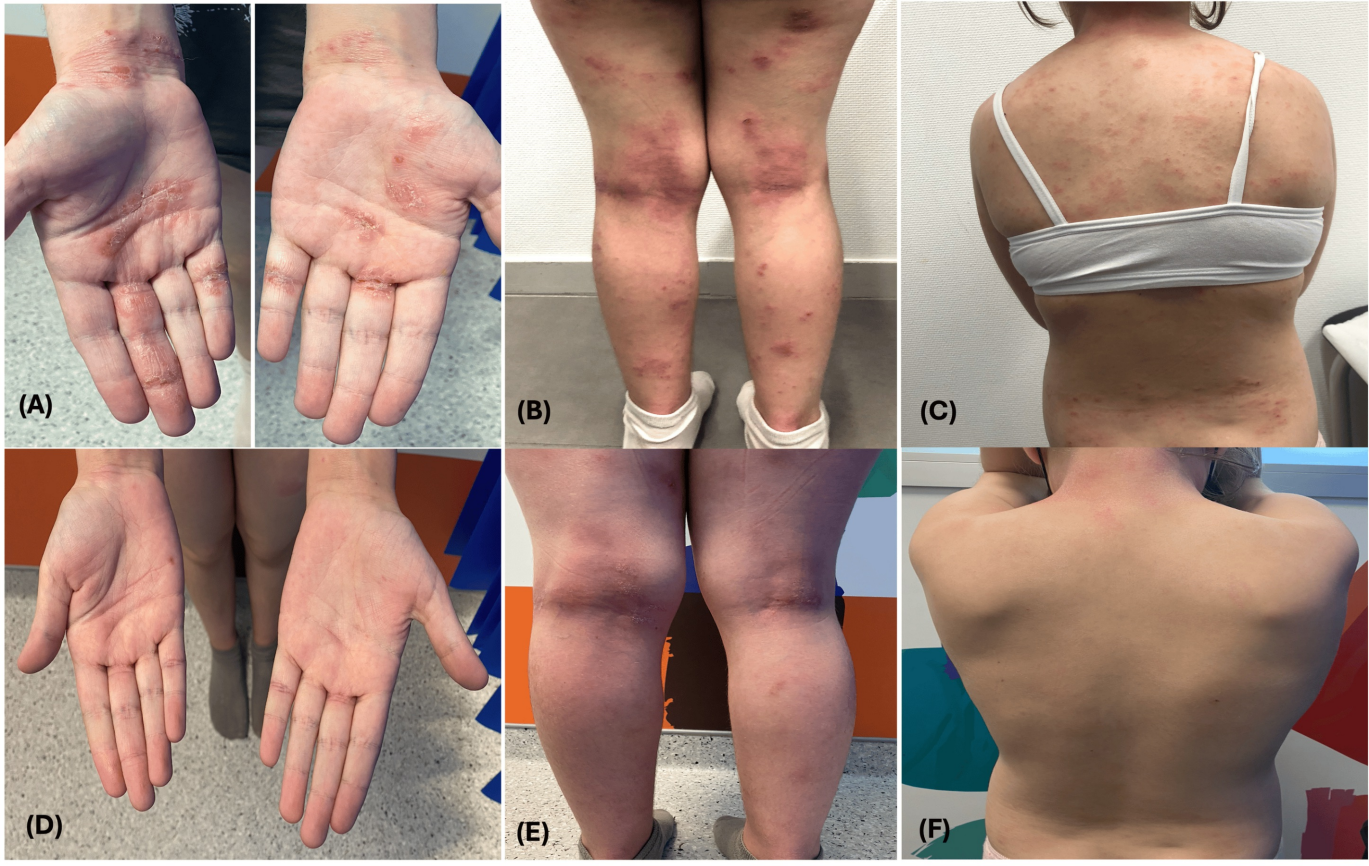
Evolution of skin lesions on the hands, legs, and back before (A, B, C) treatment with dupilumab and after (D, E, F) three months of treatment. Anonymized images included with informed parental consent.

She remains below the third percentile for height but has shown significant catch-up growth. Her standard deviation score (SDS) improved from -3.9 when she started dupilumab to -3.12 now, with a height velocity of 5.6 cm/year (SDS 2.73, P99.7). This improvement was also demonstrated by a significant decrease in allergen sensitization levels. The total IgE level is actually 889 kU/L. Clinically, she presents with mild dermatitis and almost clear skin, with a SCORAD reduction from 59.4 to 20.7 (Figure 3). 

## Discussion

A patient with pre-existing AD undergoing HSCT is at risk of developing GVHD, which can exacerbate or mimic their pre-existing skin condition. Although the pathophysiology of AD-like GVHD remains poorly understood, helper T cells (Th cells) are thought to be crucial in the development of both these conditions. The Th2 cells and their cytokines, interleukin-4 (IL-4) and interleukin-5 (IL-5), promote the production of IgE and eosinophilia, which are critical components of the atopic predisposition [6].

Dupilumab, a monoclonal antibody that inhibits IL-4 and IL-13 activity, is the first biologic with proven effectiveness and a relatively safe adverse effect profile in children and adults with Th2 inflammatory diseases and may also modulate the increased T-helper (Th)2 response described in post-transplantation [7,8].

Our case and recent literature support the use of dupilumab in transplant recipients, particularly pediatric patients with atopic AD-like GVHD. In our patient, dupilumab led to a substantial reduction in pruritus and skin lesions, along with a marked improvement in quality of life. Notably, this allowed for the gradual tapering and eventual discontinuation of systemic corticosteroids. Similar outcomes have been reported in pediatric case series by Larijani et al., Belmesk et al., and Ishee et al., in which dupilumab was effective in controlling AD-like GVHD and enabled a significant reduction in systemic immunosuppression [2,9,10]. Additionally, Tierney et al. described a child with refractory chronic cutaneous GVHD without a prior history of atopy, who showed marked clinical improvement after starting dupilumab, despite the failure of multiple conventional therapies [11]. These findings reinforce the role of dupilumab as a promising steroid-sparing option in the pediatric transplant population and support its use to minimize long-term immunosuppressive burden.

Elevated total IgE levels are frequently observed in both classic AD and AD-like GVHD, often reflecting systemic Th2 activation and allergic sensitization [6]. In our patient, initial IgE levels were markedly elevated (3040 kU/L), consistent with a severe disease phenotype and multi-organ involvement. However, in line with previous reports, these levels did not predict the therapeutic response to dupilumab. On the contrary, clinical improvement was paralleled by a progressive decline in IgE (down to 889 kU/L), supporting the systemic immunomodulatory effect of IL-4/IL-13 blockade, although this biomarker alone should not be used to guide treatment decisions [9].

In our case, the differential diagnosis prior to initiating dupilumab included classic atopic dermatitis exacerbation, drug-induced hypersensitivity, and chronic cutaneous GVHD without atopic features. A skin biopsy and multidisciplinary evaluation supported the diagnosis of AD-like GVHD, consistent with previous reports describing the histological overlap between AD and cutaneous GVHD [6,10].

This case report has several strengths, including detailed clinical documentation, use of validated disease activity scores, a multidisciplinary approach, and long-term follow-up showing both clinical improvement and reduction in corticosteroid use. Additionally, the decrease in IgE levels and catch-up growth support the systemic benefit of dupilumab beyond skin symptom control. However, limitations include the lack of a follow-up skin biopsy to confirm histological resolution and the inherent constraints of a single-patient report, which limits the generalizability of the findings.

## Conclusions

This case illustrates the diagnostic and therapeutic challenges of AD-like GVHD, particularly in pediatric patients with a history of atopy. The overlapping clinical and histological features with atopic dermatitis, combined with the burden of long-term immunosuppression, often compromise quality of life and growth. In this context, dupilumab emerged as a valuable therapeutic option, leading to marked clinical improvement, reduction in corticosteroid dependence, and enhanced quality of life.

Understandably, extended follow-up and additional larger studies are necessary to exclude any potential risks of organ rejection or changes in organ function during dupilumab treatment and to assess its long-term effectiveness and safety.
